# A qualitative exploration of reproductive coercion experiences and perceptions in four geo-culturally diverse sub-Saharan African settings

**DOI:** 10.1016/j.ssmqr.2023.100383

**Published:** 2024-06

**Authors:** Haley L. Thomas, Suzanne O. Bell, Celia Karp, Elizabeth Omoluabi, Simon P.S. Kibira, Frederick Makumbi, Hadiza Galadanci, Solomon Shiferaw, Assefa Seme, Caroline Moreau, Shannon N. Wood

**Affiliations:** a Department of Population, Family and Reproductive Health, Johns Hopkins Bloomberg School of Public Health, Baltimore, USA; b University of Western Cape, Cape Town, South Africa; c Makerere University School of Public Health, Kampala, Uganda; d Center for Advanced Medical Research and Training, Bayero University Kano, Nigeria; e Department of Reproductive Health and Health Service Management, School of Public Health, Addis Ababa University, Ethiopia; f Soins Primaires et Prévention, CESP Centre for Research in Epidemiology and Population Health, U1018, Inserm, F-94800, Villejuif, France

**Keywords:** Reproductive coercion, Contraception, Reproductive health, Qualitative research, Sub-Saharan Africa, Couples

## Abstract

Reproductive coercion (RC) is any intentional behavior that interferes with another’s reproductive decision-making or pregnancy outcome. This study aims to qualitatively examine RC experiences and perceptions among women and men in Ethiopia, Nigeria (Kano and Anambra States), and Uganda. This is a secondary analysis utilizing qualitative data from the Women’s and Girls’ Empowerment in Sexual and Reproductive Health study. Across sites, focus group discussions (38 groups; n=320 participants) and in-depth interviews (n=120) were conducted, recorded, and transcribed. Transcripts were loaded into Atlas.ti, and quotes describing experiences of reproductive control or abuse were coded as “reproductive coercion.” RC quotes were input into a matrix for thematic analysis. Emergent RC themes included indirect reproductive pressures, direct family planning interference, concurrent experiences of violence, and responses to RC. Indirect reproductive pressures included tactics to both promote and prevent pregnancy, while direct interference centered on pregnancy promotion. Women who were not compliant with their partners’ reproductive demands were often subjected to violence from multiple actors (i.e., parents, in-laws, community members) in addition to their partners. Despite concurrent forms of violence, women across sites resisted RC by using contraceptives covertly, choosing to abort, or leaving their abusive partnerships. Women and men across sites indicated that men were highly influential in fertility. RC behaviors were a mechanism of control over desired reproductive outcomes, which were often rooted in perceptions of childbearing as social status. Findings indicate a need for more nuanced community interventions targeting social norms, as well as improved RC screening and response within health services.

## 1 Introduction

Reproductive coercion (RC) is defined as any intentional behavior that interferes with another's reproductive health, autonomous decision-making, or pregnancy outcome, often to maintain power or control in a relationship (Reproductive and Sexual Coercion, 2013). RC is a direct violation of reproductive self-determination, or the right to make decisions concerning childbearing. RC encompasses many behaviors including indirect pressure or manipulation, birth control sabotage, control of a pregnancy outcome, and/or physical or sexual violence (Grace & Anderson, 2018); such behaviors can occur independently or in tandem. Though RC may co-occur with other forms of violence, such as intimate partner violence (IPV) (Miller et al., 2010; Silverman et al., 2011), it is a unique form of violence against women that has yet to be formally acknowledged by the United Nations (UN Women, 2022) despite its impact on women's health and life trajectories. RC is often perpetrated by intimate partners (Grace & Anderson, 2018), but it may also be committed or encouraged by family members (Gupta, Falb, Kpebo, & Annan, 2012; Silverman & Raj, 2014). As such, distinct perpetration patterns and motivations may be particularly poignant in low- and middle-income countries (LMICs) where intergenerational households are common (United Nations Population Division, 2019).

RC was first conceptualized by Miller and colleagues after qualitative interviews with adolescents in the United States (U.S.) indicated that pregnancy-promoting behaviors from men, such as removing a condom during sex, may explain associations between experiences of IPV and unwanted pregnancy (Miller et al., 2007, 2010). Since its conception, measures of RC have been refined, validated, and used to estimate RC prevalence, correlates, and contributors among adult populations (Grace & Anderson, 2018; McCauley et al., 2017). More recently, researchers have highlighted that the explicit intent to control reproduction is central to accurately understanding and measuring RC across the life course given that some RC behaviors, such as condom removal, may not be perpetrated with the intent to impregnate, though the behavior is violent nonetheless (Tarzia & Hegarty, 2021); this research warrants further investigation and potential quantitative measure refinement. While most research on RC has occurred within the U.S., international research on the topic is growing (Grace & Fleming, 2016; Moulton, Corona, Vaughan, & Bohren, 2021); however, findings from specific geographies, including sub-Saharan Africa (SSA), remain relatively limited.

To date, the majority of RC studies conducted in SSA are quantitative and have elucidated RC's potential health impact given observed associations with IPV (Falb, Annan, Kpebo, & Gupta, 2014), post-traumatic stress disorder (McCauley, Falb, Streich-Tilles, Kpebo, & Gupta, 2014), and covert contraceptive use (Dozier et al., 2022; Silverman, Challa, Boyce, Averbach, & Raj, 2020; Wood et al., 2020; Wood et al., 2022, Wood et al., 2022, Wood et al., 2022). The limited qualitative studies on RC in SSA highlight themes such as direct and indirect contraceptive interference, IPV in response to contraceptive use, and partner-perpetrated RC based on preferred family size (Boyce et al., 2020; Wood et al., 2020; Wood et al., 2022, Wood et al., 2022, Wood et al., 2022). Qualitative studies, however, have only been conducted in Kenya (Boyce et al., 2020; Wood et al., 2020; Wood et al., 2022, Wood et al., 2022, Wood et al., 2022). Further, all studies exploring RC in SSA have focused exclusively on women (Dozier et al., 2022; Falb et al., 2014; McCauley et al., 2014; Wood, Dozier, et al., 2022; Wood et al., 2023, Wood et al., 2023) or sub-populations of women, such as adolescent girls/young women (Decker et al., 2021; DeLong et al., 2020; Silverman et al., 2020) or women who are IPV survivors (Wood et al., 2020; Wood, Kennedy, Akumu, Tallam, Asira, Hameeduddin, et al., 2022).

There are currently no studies specific to RC in SSA that involve men, whose perspectives are critical to understanding community attitudes and acceptance of RC as well as men's motivations surrounding perpetration given their biological and social influences on women's reproductive outcomes. Research in the U.S. indicates men's perpetration behaviors may be rooted in power and control over a woman and the relationship (Alexander et al., 2021; Holliday et al., 2018). The limited studies from SSA, however, reveal that men's RC motivations may be different. Specifically, while men influence decisions regarding contraceptive use, abortion, and timing of pregnancies (Dudgeon & Inhorn, 2004), these influences are often rooted in social pressures and expectations of biological children and larger family sizes (Ngure et al., 2014) and may not be as firmly grounded in gender-based power inequities. Additional literature highlights the social consequences of childlessness (Dyer, Abrahams, Hoffman, & van der Spuy, 2002), which may provide some insight into understanding RC perpetration or attempts to control pregnancy outcomes in this context. Clarity on the dynamics of reproductive decision-making between partners is needed, particularly in settings with gendered social norms that may leave women vulnerable to RC and its adverse health effects.

The present qualitative analysis focuses on four geographically and culturally diverse sites in SSA—Ethiopia, the Kano and Anambra states of Nigeria, and Uganda. These sites have diverse social contexts with potentially differential impacts on RC perpetration and victimization. First, these countries have differing marriage practices, with Nigeria and Uganda having higher proportions of polygyny (29.9% and 27.1%, respectively) compared to Ethiopia (9.9%) (Ahinkorah, 2021). There is state-level variation within Nigeria as well, with polygyny being more prevalent in the northern states, like Kano (42%–50%), as compared to southern states, like Anambra (3%–12%) (National Population Commission NPC Nigeria and, 2019). Women in polygynous unions report wanting more children than their monogamous counterparts (Millogo, Labité, & Greenbaum, 2022), and polygyny has been shown to be associated with RC (Wood, Thomas, Guiella, et al., 2023). These countries are also currently at differing stages of fertility transition—or the demographic transition from higher to lower fertility levels—(Total Fertility Rate 4.2, 5.3 and 4.7 in Ethiopia, Nigeria, and Uganda, respectively) (World Bank Open Data, 2021) and with varying mean desired number of children (5.50 in Ethiopia, 5.99 in Uganda, and 6.49 in Nigeria) (Haque, Alam, Rahman, Keramat, & Al-Hanawi, 2021). Further, systems of kinship differ by site, which influence social order, relationships, and duty to lineage; these systems have been reported to impact fertility pressures and contraceptive practices (Caldwell, 1996). Moreover, contexts report varying lifetime prevalence of IPV (24% Nigeria, 37% Ethiopia, 45% Uganda) (WHO, 2022) and current use of modern contraceptive methods (36% Ethiopia, 28% Nigeria, 50% Uganda) (PMA, 2023), which may point to underlying differences in gender inequity, and in turn, RC behaviors and motivations. Finally, abortion legality varies by site, with Uganda and Nigeria having more restrictive abortion laws compared to Ethiopia where there are broader social and economic grounds for abortion (Center for Reproductive Rights, 2023), potentially impacting women's and men's use of abortion as either a covert act in combatting RC or coercive act in perpetrating RC.

To date, only three studies have been published on RC in these sites—one among young women in urban Lagos, Nigeria (Decker et al., 2021), and two among national samples of women in Ethiopia (Dozier et al., 2022; Wood, Dozier, et al., 2022). In Nigeria, Decker and colleagues found that transactional sex outside of a primary relationship was associated with RC (Decker et al., 2021). In Ethiopia, Wood and colleagues found that 20% of women reported past year RC, and those women had a decreased odds of modern contraceptive use (Wood, Dozier, et al., 2022). Further, Dozier et al. found that Ethiopian women experiencing RC had an increased risk of covert contraceptive use, and the proportion of covert use increased with coercion severity (Dozier et al., 2022). Given relationships between RC, contraceptive use, and IPV found in other settings, clarity is needed on the RC behaviors that women experience, as well as men's perspectives and potential motivations. To address these knowledge gaps, this secondary analysis aimed to qualitatively explore experiences or perceptions of RC among men and women in Ethiopia, Kano and Anambra states in Nigeria, and Uganda.

## 2 Methods

### 2.1 Parent study

This secondary qualitative analysis focused on RC utilizes data from the Women's and Girls' Empowerment in Sexual and Reproductive Health (WGE-SRH) study, a multi-country mixed-methods study conducted under the Performance Monitoring and Accountability 2020 (PMA2020) project. The WGE-SRH study sought to develop a framework and subsequent index for processes of empowerment specific to outcomes of pregnancy, contraceptive use, and sex by choice across diverse contexts in SSA (Moreau et al., 2018). These four distinct sites were selected to represent a range of East and West African cultures, while also leveraging existing research collaborations in those study sites for project implementation and support. Formative qualitative data collection included focus group discussions (FGDs) and in-depth interviews (IDIs) among men and women in each geography.

Qualitative data were collected between March and November 2017 in Ethiopia, the Anambra and Kano states of Nigeria, and Uganda. All study procedures were approved by Institutional Review Board (IRB) committees at Johns Hopkins Bloomberg School of Public Health, Addis Ababa University School of Public Health, Anambra Ministry of Health, Bayero University of Kano, and Makerere University School of Public Health. Participants provided informed verbal or written consent following country-specific IRB guidelines. Complete qualitative study procedures are outlined elsewhere (Karp et al., 2020, Wood et al., 2021).

### 2.2 Definition of concepts

Sex is a biological construct that is assigned to individuals at birth on the basis of their hormones, genetics, anatomy, and physiology; most individual's sex is categorized as either male or female (NIH Office of Research on Women’s Health). Gender is a social construct that links an individual's personal gender identity with their outward gender expression (NIH Office of Research on Women’s Health). Communities define gender roles and norms, and individuals face gender inequalities if their performed gender does not meet societal expectations based on those roles and norms (National Institutes of Health NIH, 2022). While sex and gender are often conflated, they are separate constructs that have different implications for individuals' health, well-being, and social standing (World Health Organization, 2021). This present study is concerned with gender and how participants' gendered social position impacts their reproductive dynamics. Participants self-reported their gender identities as either man or woman during study recruitment.

### 2.3 Focus group discussion procedures

After meeting with community gatekeepers and local organizations in one rural and one urban community per site, interviewers identified and recruited eligible FGD participants via community-based stratified purposive sampling (stratified by gender, marital status, age group, and residence). Unmarried and married women of reproductive age (15–49 years) were eligible to participate in strata-specific FGDs, while only married men (18 years or older) were eligible to participate, per study protocols. FGDs with men and women were not linked; therefore, participation by spouses in the gender-specific FGDs was not necessary.

An FGD interview guide was developed based on a preliminary WGE-SRH conceptual framework (Karp et al., 2020, Wood et al., 2021). The guide focused on community perspectives and norms regarding pregnancy and childbearing, reproductive autonomy, sexual decision-making, and family planning. In each site, trained interviewers, who were matched by gender with participants, piloted the FGD guide for context- and language-specific refinement.

FGDs were conducted in private, community buildings among groups divided by gender and age, with up to ten individuals in each group. Interviewers conducted ten FGDs in Ethiopia, Kano, and Uganda, and eight in Anambra, for a total of 38 FGDs (n = 320 participants across all sites). At each site, interviewers conducted two FGDs among young, unmarried women ages 15–17, two among married women ages 18–24, two among married women ages 30–49, and two among married men ages 18 or older. For each age group, one FGD was among urban participants and the other was among rural participants. FGDs were carried out in site-specific languages, lasted an average of 90 minutes, and were audio-recorded following participant consent. After each FGD, participants were privately screened for emotional upset and provided with a list of local resources should they feel they need support.

### 2.4 In-depth interview procedures

Eligible IDI participants were identified and recruited by in-country interviewers via community-based purposive sampling (stratified by gender, marital status, age group, and residence). Married and unmarried women of reproductive age (15–49 years) were eligible to participate in IDIs. Spouses of the married women were eligible to participate if their wife first granted permission. This permission was required for married men given the sensitive nature of the topics discussed in the interviews which could potentially elicit negative reactions among couples where both partners participated. This procedure is in line with best practices for violence-related research to protect against potential retaliation from a partner.

The multi-country study team developed, refined, and piloted the IDI guide which sought to explore individual experiences with sex, pregnancy, family planning, and decision-making. Thirty IDIs were conducted in each site (n = 120 across all sites); 24 of these were among partners (men and women interviewed separately), and six were among unmarried women ages 15–17. For each age group, half of the IDIs were among urban participants and the other half were among rural participants. IDIs were carried out in site-specific languages, lasted an average of 90 minutes, and were audio-recorded following participant consent. After each IDI, participants were screened for emotional upset and provided with a list of local resources should they feel they need support.

### 2.5 Analysis

Audio recordings of interviews were transcribed and translated into English by the interview teams; regular quality checks were performed by in-country teams to ensure accuracy of these processes. A cross-site codebook was developed based on emerging themes and used for coding transcripts in Atlas.ti, a qualitative analysis software, by the research team of the parent study. Two team members per site were involved in the coding process, alongside a central team of two coders at Johns Hopkins Bloomberg School of Public Health. The teams met jointly after every five transcripts to discuss cross-site and site-specific codes. The final coding comprised several rounds of dual coding and assessment of coding agreement. Any coding discrepancies were discussed among all team members until coding was consistent across coders and with the developed codebook. Participant quotes from the transcripts were coded as RC if they described experiences or perceptions of reproductive control or abuse, perpetrated by either a man or a woman, and inclusive of both pregnancy-promoting (i.e., taking away contraceptives) or pregnancy-preventing (i.e., forced abortion) behaviors. The code definition in Atlas.ti was “partner pressured woman into pregnancy intentions or purposely interfered with family planning use.”

For the secondary analysis, quotes coded as RC were systematically and iteratively analyzed using a matrix display (Miles, Huberman, & Saldaña, 2014). Quotes were input into rows of the matrix and tagged further with RC-specific codes (such as the perpetrator, the circumstance, or the behavior), which were then synthesized to understand the concordance of or deviation from themes among participants and across sites. Specifically, recurring codes were analyzed for convergence with other codes, and those that were similar were then grouped into broader categories. This process was repeated, and codes and categories were updated until distinct themes and sub-themes were identified. Emergent themes and sub-themes in turn became matrix columns. Illustrative quotes were marked in all relevant theme and sub-theme columns, and themes were further sorted by site, gender, age, marital status, and residence to understand convergence and divergence across sociodemographic information.

### 2.6 Reflexivity

The primary author has previous experience participating in RC research, and she came to this analysis with the belief that RC is a form of violence against women that is a violation of human rights and reproductive self-determination. She is aware that her perceptions of RC and its negative impact on women, as well as her experiences and personal values, influence her role in this work.

## 3 Results

While experiences and perceptions of RC were described across all sites regardless of gender, age, residence or marital status, the motivations or perceived motivations for RC perpetration by men were less consistent. Emergent themes classifying experiences or perceptions of RC included: 1) indirect reproductive pressures, 2) direct family planning interference, 3) concurrent experiences of violence, and 4) women's responses to resist RC, as described further below.

### 3.1 Indirect reproductive pressures

Across sites, women reported that indirect forms of RC, such as threats of separation or abandonment, were common in partnerships with discordant pregnancy intentions, or partnerships where husband and wife disagreed about the number or timing of children. Indirect reproductive pressures, such as sanctions, were used to both promote and prevent pregnancy.

#### 3.1.1 Sanctions to promote pregnancy

Loss of support or companionship was a prominent fear among women in Kano, Anambra, and Uganda. Women voiced concerns about being hated, ignored, or neglected by their partners if they refused to become pregnant. Adolescents in Kano and Uganda shared that young girls in their communities were particularly worried about being hated or uncared for if they wanted to postpone childbearing, as complete obedience was an expectation.

If she is doing it [not having children] intentionally, the husband will develop hatred for her completely because she refuses to obey him.

-Married woman aged 15–17, rural Kano, Nigeria (FGD)

If you do not agree with his decision of having a child then … this boy will be compelled to get another girl and will abandon you completely. He will hate you …

-Unmarried woman aged 15–17, rural Uganda (FGD)

Adolescents in these sites shared that girls of their age needed men's support to continue their education or maintain their livelihoods, especially if they lacked support from their families or were orphaned. Complying with their partner's demands for children, despite divergent personal desires to prevent pregnancy, was reported as a way to ensure continued financial support and attention; notably, this sub-theme focused on partner *demands* for children, rather than couple discussions.

This man can hate you for good. He will even stop giving you the help he was extending to you. In case you agree, this man will give you whatever you want, and he will treat you well and handle you with care.

-Unmarried woman aged 15–17, rural Uganda (FGD)

Fear of losing support or companionship was often exacerbated by the fear that men would seek out new romantic relationships. Starting—or threatening to start—relationships with other women could result in separation, divorce, or unwanted polygynous partnerships. Women across Kano, Anambra, and Uganda shared that these threats of relationship dissolution were common in their communities. Women could refuse to conceive or avoid pregnancy through other means and accept the separation or polygynous partnership, or they could elect to conceive to maintain their relationship.

… if a man says that you will give birth and you say otherwise, he will go and marry another wife.

-Married woman aged 20–29, urban Anambra, Nigeria (FGD)

… majority of men are interested in having many children; very few men agree with their wives to give birth to few children. They simply tell you that they need children; failure to give him, he threatens with marrying another woman so that she gives birth to many children.

-Married woman aged 25–29, urban Uganda (FGD)

A man in Uganda corroborated these behaviors and shared that, when faced with losing their marriages or being forced into polygynous arrangements, women in his community would often cede to their partners’ pregnancy demands for children.

A man can tell a woman that ‘if you do not want to produce for me children I will get another woman,’ so the woman will be pressured to produce many children to please the man and keep her marriage.

-Married man aged 18+, urban Uganda (FGD) (Karp et al., 2020)

Additionally, some men shared that childbearing was a marital obligation that was not up for negotiation. They reported having the power to force their wives out of their homes or cut off household-level financial support if they refused to oblige.

My wife is under my care. Whatever I want, she will have to abide … The reason why I got married to her was to give birth to children for me. You come to my house without having a child … you will go back to your parent’s house.

-Married man aged 18+, urban Kano, Nigeria (IDI)

Married women in Kano and Uganda described the ability of men to destabilize their lives by forcing them out of their homes or leaving them without support to manage their house or take care of their children. These destabilizing behaviors highlighted overlaps between social and economic sanctions upon failing or refusing to achieve their partner's fertility desires.

#### 3.1.2 Sanctions to prevent pregnancy

Women not only reported experiences and perceptions of coercive behaviors from men that promoted pregnancy and childbirth, but also behaviors that discouraged pregnancy and childbirth. For example, if women continued to become pregnant and bear children without their partners’ explicit consent or approval, they could be subjected to sanctions that potentially devastated their social or economic circumstances. In all sites but Anambra, women discussed their fear of being divorced, kicked out of their homes during pregnancy, or abandoned with their newborns after giving birth if having another child was considered unacceptable by her partner.

He can easily abandon the wife during labor because if he told you not to give birth and you did it, he abandons you there …

-Married woman aged 25–29, urban Uganda (FGD)

When I said to him [that] I was going to have the third child, he said “if you give birth to the third child, I will leave the house and you will care for the children.” He told me “If you give birth again, I will leave you and you will take care of them.”

-Married woman aged 30–49, urban Ethiopia (FGD)

Men confirmed the possibility of these sanctions. As described by one man in Ethiopia, the prospect of long-term security was enough to dissuade wives away from conceiving or giving birth without their partner's permission or approval.

In our community, usually the male partner has more say, and the reason is commonly females are more dependent on males even after giving birth. Sometimes, she may have even gotten pregnant without his permission. If he didn’t approve of the pregnancy, even after she gives birth, he may not even give her the basic care because she gave birth without his consent. So, since females have this fear, they leave the decision to their husbands because they will need their help later on.

-Married man aged 18+, rural Ethiopia (FGD)

In Kano and Uganda, participants shared that women in their communities were not only subjected to sanctions if they failed to prevent pregnancy in the first place, but also if they failed to terminate unwanted pregnancies upon their partners’ request.

They [partner] will say they should abort the pregnancy, and if she doesn’t agree, it can lead to divorce.

-Married woman aged 18+, urban Kano, Nigeria (FGD)

One woman in Kano said that she terminated two pregnancies and was requested by her partner to terminate a third before she sought counsel outside their partnership.

### 3.2 Direct family planning interference

Participants in Kano and Uganda reported direct family planning interference by men, such as condom sabotage and contraceptive deception, as potential outcomes of partnerships with discordant pregnancy intentions.

The only direct birth control sabotage that was mentioned multiple times in any site was condom sabotage in Uganda. Ugandan women, despite their expressed wishes to delay or prevent childbearing, reported that women must be wary of men poking holes in condoms. Participants stated that condoms may be an appropriate contraceptive method if both partners have openly communicated and have concordant desires to prevent or delay pregnancy. However, if pregnancy intentions were discordant, condom use was risky because men could grow impatient about conceiving and secretly tamper with condoms.

You cannot take a condom for granted when you have not agreed with the man because a man can put in a hole and deceive you and make you pregnant.

-Married woman aged 30–49, rural Uganda (FGD)

Fear of condom sabotage was consistently expressed among women of all ages in Uganda, however, it appeared to be amplified among young women, as they reported that their partners were often distrustful of other men and saw pregnancy as a means of control or ownership of the woman.

… he assures you that he won’t [get you pregnant], yet at the back of his mind he wants to impregnate you because he is suspicious that you have other men at school. So, I would worry a lot if we were not protected.

-Unmarried woman aged 15–17, urban Uganda (FGD)

Though rare, one man from Kano, Nigeria described contraceptive deception. Specifically, he said he had the ability to give his partner contraception without her knowledge if she wanted a child and he did not; notably, he did not report doing so.

I can decide to give her something without her knowledge that will stop her from conceiving until when I am ready.

-Married man aged 18+, rural Kano, Nigeria (IDI)

### 3.3 Concurrent experiences of violence

Explicit IPV with RC was reported among participants from Kano, Anambra, and Uganda. Specifically, emotional, physical, and/or sexual IPV was encouraged or carried out by various actors, including partners, parents/in-laws or relatives, and community members against women who refused to have children despite their partners’ desires.

#### 3.3.1 Violence from partners

When asked about the challenges of refusing to have children upon their partner's request, many women reported that physical or emotional IPV were common and occurred concurrently with their experiences of RC. Women described feeling helpless in the face of their partners' demands for children, which often resulted in retaliation in the form of IPV. Men confirmed the use of physical IPV during discussions to prevent or delay childbearing and/or their wives' direct refusal to conceive.

The other challenge is he will be insulting you all the time, beating you, and he can even decide to chase you from his home if you fail to agree. This will be to show you that he has more power than you. How can you disagree on what he wants you to do?

-Married woman aged 30–49, urban Uganda (FGD)

… the woman who is telling her husband that she no longer wants more children knows she is taking a risk. She knows that it might earn some beating if care is not taken. She also stands to be shouted at.

-Married man aged 18+, urban Anambra, Nigeria (FGD)

#### 3.3.2 Violence from parents/in-laws or other relatives

Partners were not the only reported perpetrators of RC and violence. Various other actors, such as parents or in-laws, were involved in RC perpetration in Nigeria and Uganda, specifically.

There is a problem when the woman and her husband, including her parents, do not agree [about childbearing]. Sometimes it may get to an extent that her parents will come to the house and beat her up.

-Married woman aged 15–17, rural Kano, Nigeria (FGD)

Moreover, women expressed that it was common for in-laws to insult or reject women who were unwilling to become pregnant when their partners wanted them to. While some women reached out to their parents for help during the violence, others were forcefully moved back home or were subjected to verbal abuse or bullying from other members of their husband's family.

The man himself will be the one threatening the woman that he wants to marry another wife … even his relatives join him in the blame game and advise him to take another wife instead of living in the house with a “fellow man.”

-Married man aged 18+, urban Anambra, Nigeria (FGD)

#### 3.3.3 Community influences on violence

Across sites, findings indicated that status among men was derived from control of childbearing, and any delays in childbearing could put their status into question among community members. Community gossip impacted the behaviors of men seeking to maintain or reclaim their status in society. One woman in Uganda stated that pressure to conceive from the community could be so pervasive that it could encourage sexual violence to force a pregnancy.

The community will talk ill of the couple; the man will be branded docile, and they will imagine how a woman can eat his money without anything gainful. They will continue to say that should they be in his position, they would send her away or make her pregnant by force. This may force this man to succumb to what the community says, and he acts. It would be in such a way that immediately after her menstrual period, he can force her into sex, and she conceives. Remember she will be ill prepared for this. That is what they will say and probably do.

-Unmarried woman aged 15–17, rural Uganda (FGD)

Despite women being subjected to multiple forms of violence by their partners and/or community members, shame and blame for being disobedient to their husband's fertility demands brought an additional layer of emotional abuse. A participant in Kano described a scenario in which a woman was blamed for continuing to conceive despite her husband's wish to prevent pregnancy.

I know of a woman whose husband is desperately in need of the family planning but the wife refuses. The moment she tells him that she is pregnant; he will start to beat her. Later, people begin to blame her for not being obedient to her husband.

-Unmarried woman aged 15–17, urban Kano, Nigeria (FGD)

### 3.4 Women's responses to RC

Despite these pervasive, and oftentimes violent, experiences of RC, some women across sites displayed agency in resisting their partners' behaviors. Their responses to RC largely served as means to prevent pregnancy despite their partner's wishes (through covert contraceptive use or abortion).

#### 3.4.1 Covert contraceptive use

One way that women used to take back control of their fertility in response to RC was covert contraceptive use (i.e., using contraception without their partners’ knowledge). Reports from both men and women across all four sites indicated that covert use was common, and women openly shared their thoughts or experiences about using covertly.

Sometimes women, you do not need to be very honest about the fact that you are using family planning. It is better to keep it to yourself. If he tells you to give birth … you do your thing with doctors and keep lying.

-Married woman aged 25–29, urban Uganda (FGD)

Using contraceptives covertly to resist RC, however, was often not without severe consequences. Both men and women reported that a partner's discovery of covert use could lead to abandonment or divorce.

… you may use contraceptives without the husband’s knowledge, like me. My husband was attending a training, so I went and started using contraceptives because I had a breastfeeding baby. I made a mistake of putting the family planning card in the bag, so as he was looking for something he read it, he quarreled, and told me, “It is time for you to pack up your things and go back to your home.”

-Married woman aged 18–24, urban Uganda (FGD) (Kibira et al., 2020)

She will go for family planning unknowingly to him. Some could find the packet of the medicine in the house, and sometimes it could lead to divorce.

-Married man aged 18+, urban Kano, Nigeria (FGD)

In Anambra, Ethiopia, and Uganda, participants reported that discovery of covert contraceptive use in response to RC use could also lead to physical IPV. Women in Uganda highlighted the cyclic nature of RC, IPV, and covert contraceptive use.

Few men approve the use of family planning. Often when they find pills in the house, they can overreact to the extent of beating the woman near to death. So, then the woman uses the pill stealthily without the knowledge of the husband; the same applies to injectables.

-Married woman aged 25–29, urban Uganda (FGD)

#### 3.4.2 Covert abortion

Another way women reported controlling their fertility in response to RC was through covert abortion, though this was only described by participants in Kano, Nigeria. Both men and women in Kano shared that women could abort without their partner's knowledge if they were forced to became pregnant but refused to give birth.

If he pressures her and she insists [on not giving birth], he will have to let it go … she can get rid of it [the pregnancy] without his consent.

-Married man aged 18+, urban Kano, Nigeria (FGD)

If she wants something and the husband wants another, they cannot live in peace. Some will even get pregnant and abort it.

-Married woman aged 18+, urban Kano, Nigeria (FGD)

## 4 Discussion

This secondary analysis is the first to concurrently explore women's experiences or perceptions of RC and men's motivations for RC perpetration or perceptions thereof through a multi-site qualitative study across diverse settings in SSA. Emergent themes centered on indirect reproductive pressure, direct family planning interference, concurrent experiences of violence, and women's responses to RC. For both men and women, the majority of RC narratives were shared within the context of FGDs rather than IDIs, and as such, were indicative of known or rumored anecdotes rather than sensitive, personal experiences. However, many of these reports went unchallenged by others in the groups, potentially indicating how common these experiences are. Notably, women's RC narratives prominently described sanctions or violence with discrepant fertility intentions, rather than direct contraceptive sabotage. While men's narratives centered on community norms rather than their personal experiences of perpetration, consensus indicated motivations were rooted in the desire to either control women in relationships or maintain social status within the community. Despite previous RC research primarily concentrating on men's pronatalist behaviors (DeLong et al., 2020; Dozier et al., 2022; Wood, Dozier, et al., 2022), our qualitative findings described a broader range of RC, including a multitude of ways that partners could both promote or interfere with a pregnancy, including thorough abortion coercion.

Our findings highlight that women experience many types of indirect reproductive pressures from their partners, primarily in the form of economic sanctions (i.e., discontinuation of financial support), when their partners wished to promote or prevent pregnancy. Further, men threatened separation or divorce, and new partners or polygynous marriages, all of which can destabilize women's lives. Women feared being hated, ignored, neglected, or forced out of their homes by their partners if they refused to meet their childbearing *demands*; notably, these were not just couple disagreements surrounding childbearing, but ultimatums. Though the fear of social and/or economic sanctions was common across all age groups in Nigeria and Uganda, these fears seemed of particular concern to adolescent girls and younger women who needed additional financial support. Findings on threats to withhold financial support in response to a woman's defiance of her partner's desires to become pregnant align with previous studies that have described financial dependence as a mechanism of control (Camp, 2014; Moore, Frohwirth, & Miller, 2010). These coercive dynamics prevented women from making autonomous reproductive decisions and led to compliance with their partners' demands out of fear that they would be unable to support themselves or their children if their partners were to cut ties or force them from their family home.

Results also suggest that women are at risk of violence—directly from partners and extended family members, such as parents and in-laws, and indirectly from communities—if they choose to control their pregnancy outcomes counter to the intentions of the reproductive gatekeepers in their lives, such as their partners or family members. Specifically, both men and women described the potential for emotional and/or physical violence upon refusal to have more children. Similar experiences of violence from in-laws have been documented previously in Cote d’Ivoire (Gupta et al., 2012) and have implications for mental health, as suggested by associations found between RC and probable post-traumatic stress in the same parent study (McCauley et al., 2014). Moreover, these findings highlight that social norms and community dynamics may encourage violence, including as severe as forced pregnancy incurred through sexual violence. Experiences surrounding social status and childbearing norms have been described elsewhere (Karp et al., 2020), however, the effect that compounding forms of violence have on women's reproductive health and life trajectories is largely unknown. The physical and mental health impact of violence perpetrated by extended family and community members should be further explored in SSA settings. Understanding violence perpetrated by other actors will not only help discern direct ways to mitigate abuse, but also to effectively push back on socially and culturally accepted violence.

These results have direct implications for family planning providers given described links between RC, violence, and covert contraceptive use, though women were not directly asked how providers could assist in circumstances of RC. Some women reported using contraceptives covertly to limit or space births while attempting to moderate disagreements about family planning with their partners. Covert contraceptive use to maintain peace in the household has been noted previously in the WGE-SRH parent study (Kibira et al., 2020), however, many women voiced concerns that a partner's discovery of their covert contraceptive use could put them at risk of physical IPV. Such narratives highlight the intersection of IPV and the cyclic nature of RC and covert use. Previous research in Kenya has highlighted that covert use is often in response to RC, but it can also contribute to continued RC, and even IPV, upon discovery (Wood et al., 2022). It is imperative that health care providers be aware of RC and recommend contraceptive methods that can be best optimized for covert use given a woman's circumstances and preferences.

This study is not without limitations. Foremost, this study is a secondary analysis of quotes specific to RC. The parent study was not aimed at examining RC directly, but rather issues surrounding women's and girls' empowerment in sexual and reproductive health more broadly; as such, study guides did not probe specifically on RC, motivations, and related safety strategies or responses to RC and therefore is likely not fully exhaustive of themes surrounding these issues. Further, given that RC is a sensitive topic, FGDs may not have allowed for full transparency and sharing of coercive or violent experiences; these were not intended to be an avenue to relay personal experiences but to allow participants to discuss community norms surrounding childbearing. Lastly, findings from reported experiences and perceptions are not transferable to all regions in the four study settings given the purposive sampling strategy that was used to identify and recruit participants. Those who self-select into the study may be systematically different in relation to their views and experiences of RC from those who did not choose to participate.

Future RC measurement can build from these qualitative findings that provide a more nuanced understanding of reproductive power dynamics across geographically and culturally diverse settings. Foremost, the thematic analysis identified experiences of RC that describe behaviors to encourage and discourage pregnancy, revealing that men attempted to serve as gatekeepers for childbearing regardless of the reproductive outcome. This is a unique addition to the RC literature in SSA since analyses of RC among women in this region have typically focused on coercive or forced pregnancy (i.e., pronatalist behaviors) and not the prevention of pregnancy (i.e., antinatalist behaviors). While previous literature has described forced family planning use within polygynous partnerships in Kano, Nigeria (Karp et al., 2020) and among IPV survivors in Nairobi, Kenya (Wood et al., 2020), further research is required in order to understand the coercive dynamics and extent of pregnancy-preventing behaviors in SSA, as has recently been underway in other contexts, such as Bangladesh (Pearson et al., 2023). While such behaviors constrain women's autonomy, they likely have a different impact on women's health given that women are not forced to carry a pregnancy against their will; future research must seek to better understand the health impact, including potential psychological repercussions. Further, while abortion coercion is recognized as one component of RC (Grace & Anderson, 2018) and has been studied in sub-populations of women (Hathaway, Willis, Zimmer, & Silverman, 2005; Miller et al., 2007; Moore et al., 2010; Nikolajski et al., 2015), it is not captured in the validated Reproductive Coercion Scale (McCauley et al., 2017; Miller et al., 2010). Findings from our qualitative study confirm experiences of this form of abuse in SSA, further highlighting the need for expanded quantitative measures, which have been recommended previously (Tarzia & Hegarty, 2021), that capture a more comprehensive range of RC behaviors and their severity, including coerced abortion.

Moreover, while the perspectives of men included within the present study were useful to contextualize women's RC perceptions and experiences, further research with men is needed to better understand motivations behind RC perpetration, about which little is currently known in diverse contexts (Grace & Miller, 2023; Tarzia & Hegarty, 2021). One RC study inclusive of men in SSA will not be enough to fully understand why men engage in RC behaviors. These interviews underscored that both men and women faced immense pressure to initiate childbearing. Further, the described pressure came from multiple powerful actors, including family members and the community at large, which may lead men to feel that they lack autonomy in their own reproductive behaviors. While the present research uncovered acts of explicit RC in response to this internal pressure, men's motivations may be more nuanced. Understanding men's RC motivation and internalized childbearing pressure is necessary to inform intervention development, refinement, and implementation across SSA (i.e., community norms versus couple intervention strategies). Further, continued research can inform sensitization efforts for men, which may include education- or discussion-based programs to understand and change negative gender norms, as well as couple-based programs and interventions within partnerships where reproductive goals differ.

These findings are critical for informing RC screening and response services in SSA. Reported experiences highlight concurrent types of violence (RC and physical, emotional, or sexual IPV); as such, health care providers should be prepared to screen and identify women experiencing any of these abuses according to World Health Organization (WHO) guidelines for IPV Assessment and Response (World Health Organization, 2013). Specifically, providers must be prepared to detect signs of direct or indirect contraceptive interference by an intimate partner to ensure that women are linked to safety strategies. Protocols for identifying RC during routine counseling in LMICs have been tested (Uysal et al., 2020), however, to date, have not been adopted within national protocols in SSA. While continuing to train providers on identifying reproductive abuse, continued attention must be given to offering a wide range of contraceptive options to suit women's various lifestyle and relational needs. Comprehensive contraceptive counseling can help increase method continuation for women attempting to use covertly, and in areas where health services are not consistently available or are inaccessible, community-based care models should be expanded to reach women experiencing RC.

## 5 Conclusions

RC is a violation of human rights and justice and is often perpetrated in settings with patriarchal social norms and values. Across the four study sites in SSA, men and women reported similar views on RC. Findings illustrate that some men readily interfere in women's reproductive decision-making, indicating a need for more nuanced community interventions, including those involving men, as well as increase RC screening and response services within the health sector.

## CRediT authorship contribution statement

**Haley L. Thomas:** Formal analysis, Writing – original draft, Writing – review & editing. **Suzanne O. Bell:** Writing – original draft, Writing – review & editing, Supervision. **Celia Karp:** Conceptualization, Formal analysis, Investigation, Methodology, Writing – review & editing. **Elizabeth Omoluabi:** Investigation, Methodology, Project administration, Validation, Writing – review & editing. **Simon P.S. Kibira:** Investigation, Methodology, Project administration, Validation, Writing – review & editing. **Frederick Makumbi:** Investigation, Methodology, Project administration, Validation, Writing – review & editing. **Hadiza Galadanci:** Investigation, Methodology, Project administration, Validation, Writing – review & editing. **Solomon Shiferaw:** Investigation, Methodology, Project administration, Validation, Writing – review & editing. **Assefa Seme:** Investigation, Methodology, Project administration, Validation, Writing – review & editing. **Caroline Moreau:** Conceptualization, Funding acquisition, Investigation, Methodology, Project administration, Supervision, Writing – review & editing. **Shannon N. Wood:** Conceptualization, Formal analysis, Investigation, Methodology, Project administration, Supervision, Validation, Writing – original draft, Writing – review & editing.

## Declaration of competing interest

The authors declare that they have no known competing financial interests or personal relationships that could have appeared to influence the work reported in this paper.

## List of abbreviations

RC: Reproductive coercion
IPV: Intimiate partner violence
LMIC: Low- and middle-income country
U.S.: United States
SSA: Sub-Saharan Africa
WGE-SRH: Women's and Girls' Empowerment in Sexual and Reproductive Health
PMA2020: Performance Monitoring and Accountability 2020
FGD: Focus group discussion
IDI: In-depth interview
IRB: Institutional Review Board
WHO: World Health Organization
